# Cervical Squamous Intraepithelial Lesions Are Associated with Differences in the Vaginal Microbiota of Mexican Women

**DOI:** 10.1128/Spectrum.00143-21

**Published:** 2021-10-13

**Authors:** M. E. Nieves-Ramírez, O. Partida-Rodríguez, P. Moran, A. Serrano-Vázquez, H. Pérez-Juárez, M. E. Pérez-Rodríguez, M. C. Arrieta, C. Ximénez-García, B. B. Finlay

**Affiliations:** a Michael Smith Laboratories, Department of Microbiology and Immunology, University of British Columbia, Vancouver, British Columbia, Canada; b Laboratorio de Inmunología, Unidad de Investigación de Medicina Experimental, Facultad de Medicina, Universidad Nacional Autónoma de México, Mexico City, Mexico; c Department of Physiology and Pharmacology, Cumming School of Medicine, University of Calgary, Calgary, Alberta, Canada; d Department of Pediatrics, Cumming School of Medicine, University of Calgary, Calgary, Alberta, Canada; e Laboratorio de Inmunología, UAEM Pediatría del Centro Médico Nacional SXXI, Instituto Mexicano del Seguro Social, Mexico City, Mexico; f Department of Microbiology and Immunology, University of British Columbia, Vancouver, British Columbia, Canada; g Department of Biochemistry and Molecular Biology, University of British Columbia, Vancouver, British Columbia, Canada; Lerner Research Institute

**Keywords:** 16S rRNA gene, HPV, squamous intraepithelial lesions, vaginal microbiota

## Abstract

Cervical cancer is an important health concern worldwide and is one of the leading causes of death in Mexican women. Previous studies have shown changes in the female genital tract microbe community related to human papillomavirus (HPV) infection and cervical cancer; yet, this link remains unexplored in many human populations. This study evaluated the vaginal bacterial community among Mexican women with precancerous squamous intraepithelial lesions (SIL). We sequenced the V3 region of the 16S rRNA gene in cervical samples from 228 Mexican women, including 121 participants with SIL, most of which were HPV positive, and 107 healthy women without HPV infection or SIL. The presence of SIL was associated with changes in composition (beta diversity) and with a higher species richness (Chao1). A comparison of HPV-positive women with and without SIL showed that microbiota changes occurred even in the absence of SIL. Multivariate association with linear models (MaAsLin) analysis yielded independent associations between HPV infection and an increase in the relative abundance of Brachybacterium conglomeratum and Brevibacterium aureum as well as a decrease in two Lactobacillus iners operational taxonomic units (OTUs). We also identified a positive independent association between HPV-16, the most common HPV subtype linked to SIL, and Brachybacterium conglomeratum. Our work indicates that HPV infection leading to SIL is primarily associated with shifts in vaginal microbiota composition, some of which may be specific to this human population.

**IMPORTANCE** Human papillomavirus (HPV) plays a critical role in cervical carcinogenesis but is not sufficient for cervical cancer development, indicating the involvement of other factors. The vaginal microbiota is an important factor in controlling infections caused by HPV, and, depending on its composition, it can modulate the microenvironment in vaginal mucosa against viral infections. Ethnic and sociodemographic factors influence differences in vaginal microbiome composition, which underlies the dysbiotic patterns linked to HPV infection and cervical cancer across different populations of women. Here, we provide evidence for associations between vaginal microbiota patterns and HPV infection linked to ethnic and sociodemographic factors. To our knowledge, this is the first report of the species *Brevibacterium aureum* and Brachybacterium conglomeratum linked to HPV infection or squamous intraepithelial lesions (SIL).

## INTRODUCTION

Cervical cancer is one of the most common cancers and one of the leading causes of death in women worldwide (1, 2). Cervical cancer is causally related to human papillomavirus (HPV) infection, an oncogenic virus actively involved in cervical epithelium transformation (3, 4). After HPV infection and persistency, squamous intraepithelial lesion (SIL) development may occur, which may heal or persist and evolve to cancer (1). Despite overwhelming evidence that certain subtypes of HPV are the main causative agents of SIL development and progression to cervical cancer, it is also well established that HPV alone is not sufficient to induce cervical malignant transformation (4–7). Many factors have been associated with the appearance of SIL, such as intermenstrual bleeding, multiparity, use of contraceptives, multiple sexual partners, and smoking (8).

In addition to these variables, it has been proposed that the vaginal microbiota plays an important role in the development of HPV infection leading to cervical neoplasm (9). This is aligned with the endorsed concept in infection biology, in which successful pathogen colonization and infection embodies dynamic interactions between the infecting microbes, host factors, and the microbiome (10). The vaginal microbiota is a complex microbial ecosystem influenced by environmental and host factors as well as ethnic background (11). The vaginal microbiota in healthy women consists of over 200 bacterial species, but this ecosystem is generally dominated by *Lactobacillus* spp. Lactobacilli provide broad-spectrum protection by producing lactic acid, bacteriocins, and biosurfactants and by adhering to the mucosa that forms barriers against pathogenic infections in the vaginal microenvironment (2, 12). Following imbalance of this defense system, physicochemical changes arise, inducing histological alterations of the vaginal mucosa and the cervical epithelium, all of which put a selective pressure on the microbiota (13–15).

Some vaginal microorganisms, such as *Gardnerella*, *Fusobacteria*, Bacillus cohnii, *Dialister*, *Prevotella*, and *Mycoplasma*, as well as a decrease in the proportion of *Lactobacillus* spp. have been linked to dysbiosis that would generate an unstable microenvironment, which in turn could enable the effect of key risk factors in cervical cancer (16–19). Some of these changes are responsible for increasing the levels of mucin-degrading enzymes, which may play a role in the degradation of the mucous layer that covers the vaginal and cervical epithelium and endocervical mucus (20, 21). There is evidence of HPV evasion or infection mechanisms that support that microorganisms such as *Sneathia*, *Anaerococcus*, *Fusobacterium*, and *Gardnerella* are implicated with higher frequency and severity of disease, potentially resulting in precancerous and cancerous cervical lesions (22).

However, these findings are not uniform across studied populations. Despite the fact that Latin American countries have a high prevalence of HPV and cervical cancer is one of the main causes of death in Latin American women (3, 23–25), including Mexico (7, 9), most of the studies have been conducted in industrialized countries (26). Likewise, the projected demographic changes in Latin America imply that the current burden of new cervical cancer cases will increase in the next 20 years (2, 27). The evidence observed so far suggests that the ethnic and sociodemographic factors that influence differences in vaginal microbiota composition may also underlie dysbiotic patterns linked to HPV infection and cervical cancer across different Latin American women populations (3, 7, 9, 23–25). Therefore, there is a growing need for more evidence on the association between vaginal microbiota patterns and HPV infection in Latin America and its relationship with the progression of SIL to cervical cancer.

In this work, we compared the vaginal microbiota in 228 Mexican women with precancerous SIL to those of healthy controls, while taking into consideration the confounding effects of clinical-, behavioral-, and HPV infection-related variables, the type of premalignant cervical lesion, and the genetic variants of the virus.

## RESULTS

### Study participant characteristics: cases versus controls.

A total of 228 samples were analyzed, consisting of 107 controls and 121 cases. Of the 121 cases, 90 were diagnosed with low squamous intraepithelial lesion (LSIL) (women diagnosed with HPV infection and cervical intraepithelial neoplasia 1 [CIN1]), and 31 were diagnosed with HPV infection and high squamous intraepithelial lesion (HSIL) (women diagnosed with CIN2 or CIN3). All women were cancer free, but cases exhibited altered results in cytological, histological, and colposcopy analyses, and control, healthy women exhibited normal results.

By molecular analysis, the HPV subtype frequency was highest for HPV-16, HPV-58, and HPV-18 types, with 53.72%, 15.7%, and 9.1%, respectively, observed within cases. Within women without SIL, 32.7% were positive for HPV by PCR, and the distribution of HPV subtypes was similar to that observed in cases. Most of the women in both groups did not smoke (75.21% of cases versus 71.96% of controls), had regular menstrual periods (69.42% of cases versus 68.22% of controls), and most did not have intermenstrual bleeding (85.95% of cases versus 88.79% of controls). Statistically significant differences between groups were detected in relation to an active sexual life at the time of the study (73.55% of cases versus 92.53% of controls), use of contraceptives (66.12% of cases versus 52.34% of controls), and genital hygiene, recorded by the frequency of vaginal douching (81.82% of cases versus 52.34% of controls). More details of the characteristics of each group are described in Table 1. When comparing continuous variables, cases and controls differed by age (37.26 ± 10.87 years in cases versus 42.83 ± 7.92 years in controls) and age of sexual debut (age 18 interquartile range [IQR] of 4 in cases versus age 20 IQR of 5 in controls). There were also significant differences in the number of births and miscarriages, but these values are likely skewed by age differences between the two groups (Table 2).

**TABLE 1:** Characteristics of the study population (SIL-positive and SIL-negative participants): categorical variables

Variable	Subcategory	SIL positive (*N* = 121)	SIL negative (*N* = 107)	*P* ^*a*^ ^,^ ^*b*^	OR^*b*^	CI^*b*^
SIL grade	Low grade	90 (74.38%)	0	NA	NA	NA
	High grade	31 (25.62%)	0			
HPV	Positive	121 (100%)	35 (32.7%)	**< 2.2E−16** ^*c*^	2,548.8^*c*^	61.81 to 22,163.8^*c*^
	Negative	0	72 (100%)			
HPV type	HPV 16	65 (53.72%)	22 (20.56%)	**< 2.2E−16** ^*c*^	NA	NA
	HPV 58	19 (15.70%)	6 (5.61%)			
	HPV 18	11 (9.1%)	2 (1.86%)			
	HPV 31	5 (4.13%)	1 (0.09%)			
	HPV 11	4 (3.31%)	0			
	HPV 45	4 (3.31%)	1 (0.09%)			
	Other	13 (10.7%)	3 (2.8%)			
	HPV Neg	0	72 (67.2%)			
Smoking	Yes	31 (25.62%)	30 (28.04%)	0.6807	0.8841	0.49 to 1.59
	No	90 (75.21%)	77 (71.96%)			
Menstrual period	Regular	84 (69.42%)	73 (68.22%)	0.8455	0.946	0.54 to 1.66
	Irregular	37 (30.58%)	34 (31.76%)			
Intermenstrual bleeding	Yes	17 (14.05%)	12 (11.21%)	0.5215	1.29	0.59 to 2.85
	No	104 (85.95%)	95 (88.79%)			
Sexually active (at study assessment)	Yes	89 (73.55%)	99 (92.52%)	**0.000171**	0.225	0.098 to 0.513
	No	32 (24.45%)	8 (7.48%)			
Use of contraceptive(s)	Yes	80 (66.12%)	56 (52.34%)	**0.0343**	1.777	1.041 to 3.032
	No	41 (33.88%)	51 (47.66%)			
Contraceptive type	IUD	30 (24.79%)	15 (14.02%)	**0.00044** ^*c*^	NA	NA
	Tubal ligation	22 (18.18%)	13 (12.15%)			
	Hormonal	16 (13.22%)	9 (8.41%)			
	Condom	9 (7.44%)	2 (1.87%)			
	Other	3 (2.48%)	5 (4.67%)			
	Did not specify	0	13 (12.15%)			
	None	41 (33.88%)	50 (46.73%)			
Vaginal douching	Yes	22 (18.18%)	51 (47.66%)	**1.918E−06**	0.244	0.134 to 0.444
	No	99 (81.82%)	56 (52.34%)			

a *P* values in bold denote statistical significance (*P* > 0.05).

b NA, not available.

c Denotes value after Yates continuity correction.

**TABLE 2:** Characteristics of the study population (SIL-positive and SIL-negative participants): continuous variables

Variable	SIL positive (*N* = 121)^*a*^	SIL negative (*N* = 107)^*a*^	Normality test (D'Agostino and Pearson)	*P* ^*b*^
Age, yrs	37.26 ± 10.87	42.83 ± 7.92	Normal	**1.308E−05**
Age since sexually active	18 (IQR = 4)	20 (IQR = 5)	Nonnormal	**3.559E−05**
No. of sexual partners	1 (IQR = 1)	1 (IQR = 1)	Nonnormal	0.9955
No. of pregnancies	3 (IQR = 2)	2 (IQR = 1)	Nonnormal	0.3498
No. of births	2 (IQR = 2)	2 (IQR = 2)	Nonnormal	**0.0279**
No. of miscarriages	0 (IQR = 1)	0 (IQR = 1)	Nonnormal	**0.04843**

a Mean ± standard deviation (SD) or median values and IQR based on D’Agostino and Pearson normality test.

b *P* values in bold denote statistical significance (*P* > 0.05).

### Associations between the vaginal microbiota SIL status.

**FIG 1:**
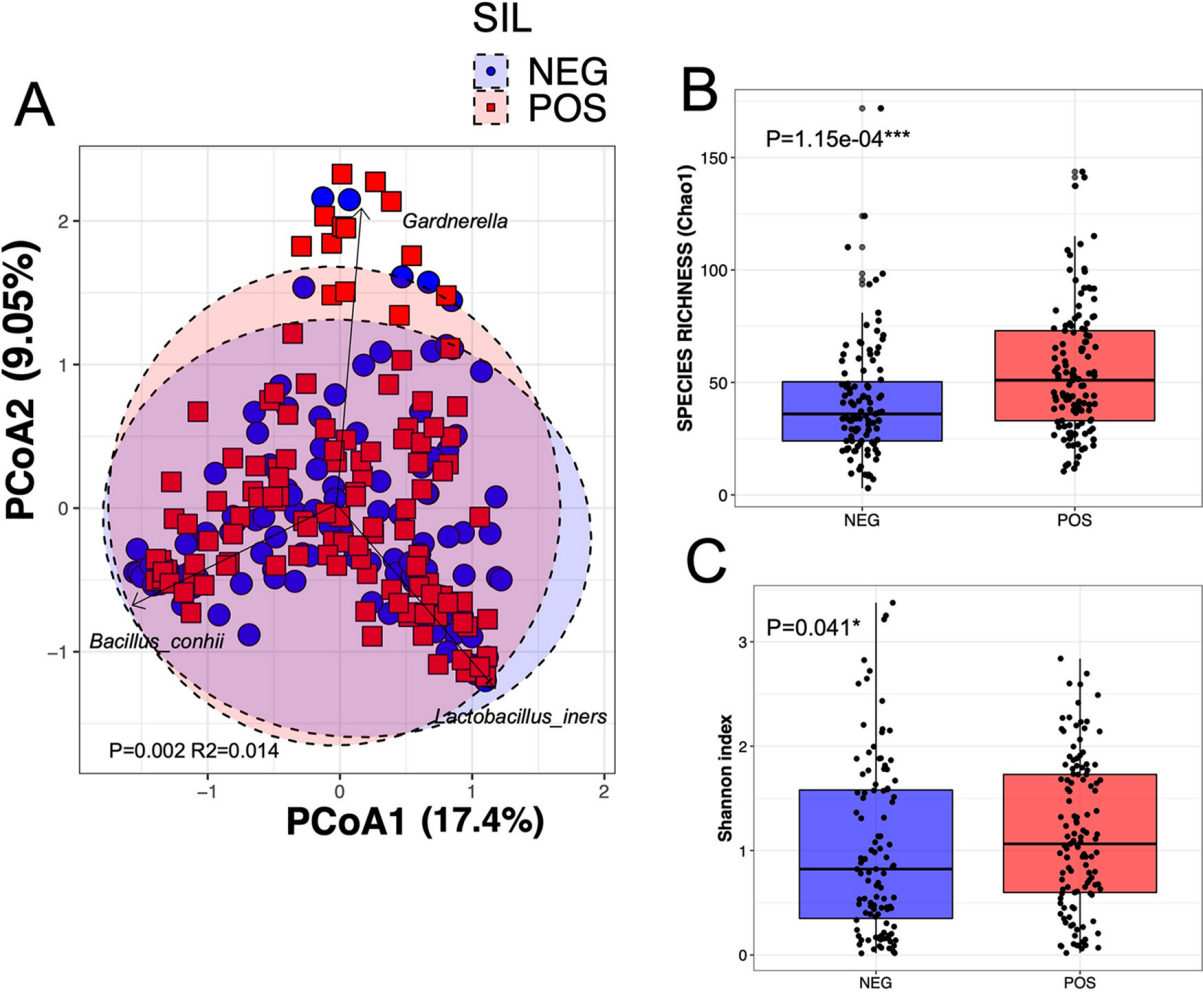
(A) Principal-coordinate analysis (PCoA) ordination of variation in beta diversity of human cervical bacterial communities in adult Mexican women based on Bray-Curtis dissimilarities. Color and shape represent the presence of squamous cervical intraepithelial lesions (SILs; blue circles represent the absence of SILs, and red squares represent the presence of SILs). PERMANOVAs indicate that the SILs represent 1.4% of the variation in vaginal bacterial community structure (*N* = 228; Adonis *P* = 0.002). Arrows represent loading plot coordinates for the three most abundant OTU features in the data set; NEG, negative; POS, positive. Variation in species richness (B) (Chao1) and alpha diversity (C) (Shannon index) of vaginal bacterial communities between women with (red) and without (blue) SILs. Stars denote statistical significance (*N* = 228; Mann-Whitney test).

**FIG 2:**
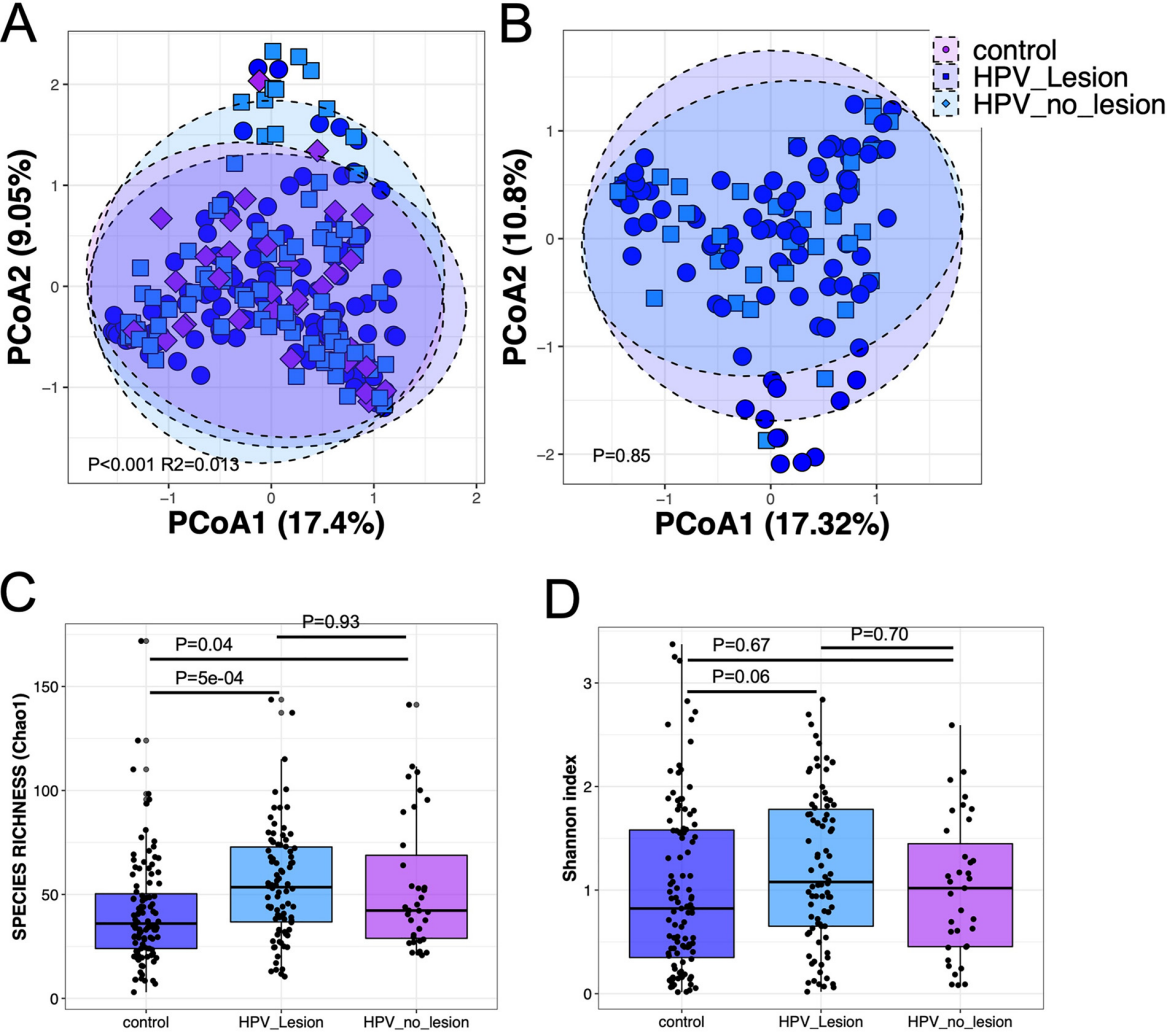
(A) Principal-coordinate analysis (PCoA) ordination of variation in beta diversity of human cervical bacterial communities in adult Mexican women based on Bray-Curtis dissimilarities. Color and shape represent presence of HPV infection with or without squamous cervical intraepithelial lesions (purple diamonds represent the absence of SILs or HPV infection, blue circles represent the presence of SILs and HPV infection, and light blue squares represent the absence of SILs in HPV-positive samples). PERMANOVAs indicate 1.3% of the variation in vaginal bacterial community structure (*N* = 228; Adonis *P* < 0.001). (B) When the two HPV-positive groups were compared, no significant variation in community composition was detected. Variation in species richness (C) (Chao1) and Shannon index (D) of vaginal bacterial communities between the three groups. *P* values of <0.05 denote statistical significance (*N* = 228; Kruskal-Wallis and Dunn tests).

We determined the bacterial community by amplification and sequencing of the 16S rRNA gene (V3 region). The presence of SILs was associated with changes in bacterial alpha and beta diversity (Fig. 1), with notable compositional differences at the family and genus level (Fig. 2). Beta diversity analysis, measured by principal-coordinate analysis (PCoA; Bray Curtis distance) (Fig. 1A) indicated that cervical SILs explain 1.4% of the variation in vaginal bacterial community structure (*N* = 228; Adonis *P* = 0.002). The presence of SILs was also associated with significantly higher species richness than an absence of SILs (Chao1; *P* = 1.15E−04) (Fig. 1B) as well as a smaller but still significant increase in the Shannon index, which considers both richness and evenness (*P* = 0.041) (Fig. 1C), suggesting that the broadest diversity change is explained by bacterial community richness.

**FIG 3:**
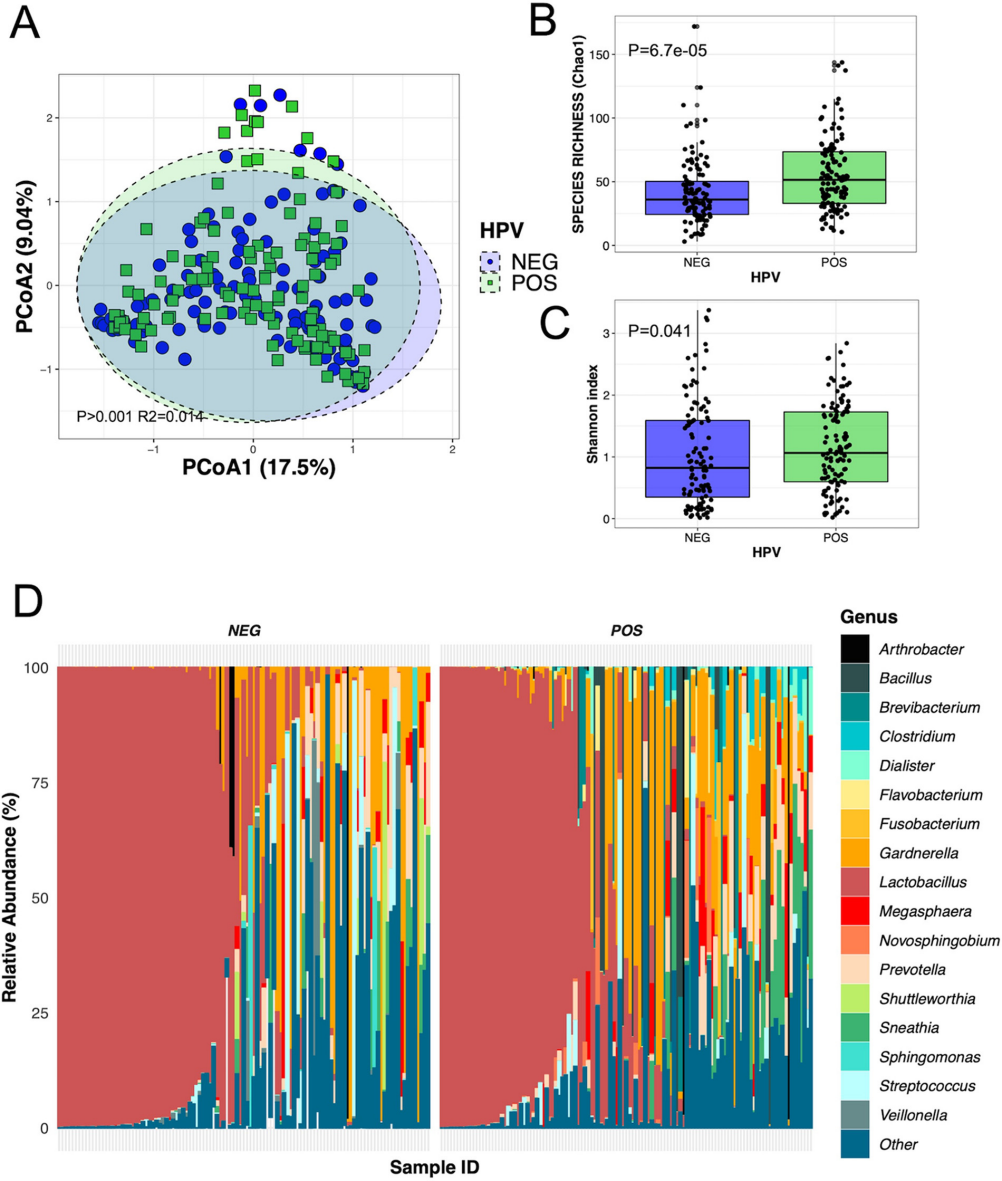
(A) Principal-coordinate analysis (PCoA) ordination of variation in beta diversity of human cervical bacterial communities in adult Mexican women based on Bray-Curtis dissimilarities. Color and shape represent the presence of HPV infection. PERMANOVAs indicate 1.4% of the variation in vaginal bacterial community structure (*N* = 228; Adonis *P* < 0.001). Variation in species richness (B) (Chao1) and species richness and evenness (C) (Shannon index) of vaginal bacterial communities between women with (green) and without (blue) HPV (*N* = 228; Mann-Whitney test). (D) Relative abundance at the genus level of individual samples with or without HPV infection included in the study (a cutoff of 1% relative abundance was applied; all taxa of <1% or without genus-level taxonomy were grouped as ‘Other’; *N* = 228).

Within this study, we identified 35 participants who had not developed SILs, yet were HPV positive. This allowed us to compare the association between HPV infection and an absence of SILs. Interestingly, beta diversity, measured by principal-coordinate analysis, detected a very similar proportion of variation in composition to that detected for SIL status (Fig. 2A). Moreover, there were no differences in beta or alpha diversities between HPV-positive samples with SILs and samples without SILs (Fig. 2B to D), suggesting that the detected vaginal microbiota shifts are primarily associated with HPV infection, which, in many cases, subsequently leads to SILs. To further investigate the effect of SILs on the vaginal microbiota, we also compared samples from women with high-grade SILs with samples from women with low-grade SILs and did not detect any differences in alpha or beta diversity (Mann-Whitney *P* = 0.41; Adonis *P* = 0.11), further strengthening the link between HPV infection, rather than SILs, on the vaginal microbiota. Because of this, we further investigated the differences in vaginal microbiota explained by HPV infection. While results are similar to when data were stratified by SIL (Fig. 1), the differences in species richness (Chao1) were more pronounced (Fig. 3A to C). Relative abundance plots of the individual samples showed that while there is great interindividual variability, there are noticeable differences between women with and without HPV infection that align with the observed increase in species richness detected by Chao1 in women with SILs and/or HPV infection (Fig. 3D).

To study associations between specific taxa, SILs, and HPV status, given the importance of controlling for potential confounding variables that could explain or correlate with the detected associations between SILs or HPV status and the microbiota, we used multivariate association with linear models (MaAsLin). MaAsLin is a multivariate linear modeling tool with boosting that tests for associations between specific microbial taxa and continuous and/or Boolean metadata. This method reduces the total amount of correlations to be tested, therefore allowing for improvements in the robustness of the additive general linear models. With MaAsLin, we found significant independent associations between HPV-positive status and an increase in the relative abundance of Brachybacterium conglomeratum and *Brevibacterium aureum* as well as a decrease in two Lactobacillus iners operational taxonomic units (OTUs) (Table 3). This indicates that no other variable, including SILs, explained the taxonomic differences influenced by HPV infection status. Interestingly, other independent associations were also detected between HPV subtypes or contraception use and several bacterial taxa (Table 3). For example, we detected a positive association between Brachybacterium conglomeratum and HPV 16, the most common HPV subtype associated with SILs, further suggesting the link between this bacterial species and HPV infection (Table 3).

**TABLE 3:** Differential OTUs in relation to study variables (MaAsLin)

Variable	Feature^*a*^	Ref. value^*b*^	Coefficient	*P* value	*Q* value
Contraception	G_*Fusobacterium*_Otu105	IUD	0.039	3.83E−07	3.83E−07
Contraception	S_Acinetobacter*_lwoffii*_Otu127	IUD	0.066	2.31E−06	2.31E−06
HPV_type	S_*Brachybacterium_conglomeratum*_Otu28	hpv 16	0.036	4.12E−07	4.12E−07
HPV_type	F_*Phyllobacteriaceae*_Otu152	hpv 18	−0.034	6.13E−06	6.13E−06
HPV_type	G_*Dialister*_Otu213	hpv 31	0.018	1.08E−06	1.08E−06
HPV_type	F_*Phyllobacteriaceae*_Otu152	hpv 31	−0.038	3.07E−05	3.07E−05
HPV_type	F_ S24_7_Otu103	hpv 31	0.038	3.07E−05	3.07E−05
HPV_type	F_*Phyllobacteriaceae*_Otu152	hpv 45	−0.039	1.50E−05	1.50E−05
HPV_type	S_*Prevotella_copri*_Otu24	hpv 45	0.134	2.73E−05	2.73E−05
HPV_type	S_*Lactobacillus_iners*_Otu136	hpv 53	0.063	2.31E−09	2.31E−09
HPV_type	S_*Lactobacillus_iners*_Otu180	hpv 56	0.076	7.15E−18	7.15E−18
HPV_type	F_*Phyllobacteriaceae*_Otu152	hpv 58	−0.037	4.56E−07	4.56E−07
HPV_type	G_*Clostridium*_Otu31	hpv 6	0.167	1.59E−05	1.59E−05
HPV_type	S_*Lactobacillus_iners*_Otu101	hpv 83	0.101	9.34E−06	9.34E−06
HPV_type	G_*Mycoplasma*_Otu46	hpv 90	0.585	2.35E−36	2.35E−36
HPV	S_*Lactobacillus_iners*_Otu136	POS	0.034	1.34E−06	1.34E−06
HPV	S_*Brachybacterium_conglomeratum*_Otu28	POS	0.149	3.30E−06	3.30E−06
HPV	S_*Brevibacterium_aureum*_Otu14	POS	0.348	1.45E−05	1.45E−05
HPV	S_*Lactobacillus_iners*_Otu180	POS	−0.172	1.19E−05	1.19E−05

a Features organized in ascending order of adjusted *P* values by variable.

b POS, positive; Ref, reference.

## DISCUSSION

Several factors are known to play a role in cervical carcinogenesis, with HPV infection being one of the most important in the development of the disease (1). There are more than 100 types of HPV, of which at least 14 high-risk HPV types have been defined as carcinogenic (28). In this study, we found that over half of HPV infections are caused by the HPV-16 type, followed by HPV-58 and HPV-18, and all of them are considered high-risk HPVs worldwide (29). This predominance of the HPV-16 type was expected because it is generally accepted that HPV-16 is the major high-risk genotype in Mexico and in the world (30, 31). We also found HPV-58 as the second most prevalent genotype in 15.7% of the cases, which is aligned with what has been reported in Asia (14.36 to 15.90%) (31).

Our study also revealed several other factors associated with SIL status, some of which reaffirm previously reported links (32). Factors positively associated with SILs included younger age, HPV infection, younger age of sexual debut, and the use of contraceptives, with the biggest difference explained by intrauterine device (IUD) use. In contrast, being sexually active at the time of the study and vaginal douching were linked to a reduced risk of SILs in this group of women.

Regarding contraceptive use, our results showed that an increased risk of SILs was linked to IUD use, which differs from that reported by Cortessis et al. (33) where they indicated that invasive cervical cancer can be approximately 30% less frequent in women who have used an IUD. Likewise, Agenjo et al. (34) described an inverse relationship between IUD use and cervical cancer risk, with women using IUDs reporting half the risk of developing this type of cancer. Our contrasting results, however, are in line with previous microbiome correlations with cervical cancer. We found significant correlations with IUD use and the increased abundance of Acinetobacter lwoffii, which has been previously reported in HPV-positive women (35). In addition, we detected an independent positive correlation with the use of IUDs and *Fusobacterium* sp. *Fusobacterium* has been studied as a possible diagnostic biomarker of cervical cancer because it is positively correlated with tumor differentiation (36). Furthermore, *Fusobacteriaceae* have been reported as the most abundant microorganisms in cervical carcinoma (37). Future work should study how IUD use may modulate the associations between the vaginal microbiota and SILs or cervical cancer.

While it is unclear why younger age was linked to SILs in our study, it is likely that it relates to the common age of onset of SILs, which occurs between 25 and 35 years of age (38, 39). In contrast, healthy women would be less likely to visit the Instituto Mexicano del Seguro Social (IMSS) for a routine gynecological visit. Our microbiome results did not find any differences associated with age, suggesting that age did not confound our results. Several study variables linked sexual activity with SILs, including younger age of sexual debut. These and other related sexual behavioral factors have been previously linked with SILs, HPV infection, and cervical cancer risk (40). Interestingly, our study revealed that vaginal douching was linked to a reduced risk of SILs (odds ratio [OR] of 0.24; confidence interval [CI] of 0.13 to 0.44). Studies on cervical cancer and vaginal douching have reported positive, negative, and no associations (41). Although it is unlikely that SILs would lead to symptoms that would motivate genital douching, this practice is more common among women with other risk factors linked to sexually transmitted infections, which are a common cause of symptoms.

An important finding from our study is that the vaginal microbiota differences were primarily attributed to HPV infection (or subtype) and not SILs, indicating that infection itself may lead to changes in the vaginal microbial community. Among the predominant components of a healthy vaginal microbiome is the presence of *Lactobacillus* species, including L. crispatus, *L. iners*, L. jensenii, and L. gasseri (17, 42), which results in reduced community diversity. Indeed, bacterial richness increases as *Lactobacillu*s spp. levels are reduced in association with precursor lesions of cervical cancer (17) and with HPV infection itself (2, 43, 44). In support of this, our results showed higher species richness in cases as well as shifts in beta diversity. Compositional differences involved several taxa, including lactobacilli. Two *L. iners* OTUs were decreased in women with HPV infection. *L. iners* has been previously associated with a dysbiotic community and displays a series of characteristics that make this species different from other known vaginal lactobacilli (45–47). For instance, *L. iners* is a lower producer of d-lactic acid and induces interleukin-8 (IL-8) secretion, causing proinflammatory activity in the cervix, which may influence the progression of cervical intraepithelial neoplasia (15). In other studies, the dominance of *L. iners* and interactions with other vaginal anaerobic microorganisms alters the balance of the vaginal microbiota in association with cervical intraepithelial neoplasia (13).

The most discriminant microbial differences between cases and controls involved *Brevibacterium aureum* and Brachybacterium conglomeratum (increased in cases). While these differences were very significant, these species were not uniformly present among either group (Fig. 3), suggesting that interindividual compositional differences may prevent the identification of microbiota species with biomarker potential for HPV infection or SILs. However, our study identified Brachybacterium conglomeratum as independently associated with HPV and with HPV-16, the most common subtype detected in our study. This finding prompts future investigation of the link between this bacterial species and SILs or cervical cancer risk associated with this specific HPV subtype and raises the possibility that microbiome links with HPV infection are subtype specific. To our knowledge, this is the first time this species has been linked to HPV infection. *B. conglomeratum* has not been readily reported in vaginal microbiome studies, which have surveyed mainly North American and European populations (48–50). This finding underlines the importance of considering ethnicity- and geography-driven differences in human microbiome studies, as dysbiotic patterns may be population specific.

## MATERIALS AND METHODS

### Study design.

Healthy women and women infected with HPV, regardless of the degree of cervical squamous lesion, over 18 years of age (adult age in Mexico) and attending the Instituto Mexicano del Seguro Social (IMSS) in Mexico City were invited to participate as volunteers in this study. Written informed consent was obtained from all volunteers after providing detailed information about the study and its characteristics. The clinical research protocol and letter of informed consent were evaluated and approved by the Comité Local de Investigación y de Bioética de la División de Educación e Investigación Médica de la Unidad de Alta Especialidad Médica Pediatría del IMSS. All participants completed a study questionnaire that was used to obtain sociodemographic and risk factor information. Data were registered in a secured database for subsequent statistical analysis.

A total of 228 Mexican women over 21 years of age who attended the IMSS from December 2003 to July 2006 were enrolled in the study. These women were divided in two groups. The first group (controls) consisted of 107 healthy women with a mean age of 42.8 years (±7.9) with three previous Papanicolaou (Pap) tests negative for HPV infection for three consecutive years (a fourth negative Pap result occurred at the time participants were invited to join the study) and diagnosed without SIL with normal cytology and colposcopy results by the treating gynecologist. The second group (cases) consisted of 121 patients with a mean age of 37.3 years (±10.9) with different degrees of SILs and were positive for HPV infection based on cytology, histology, and colposcopy examination. This group included women diagnosed with cervical intraepithelial neoplasia from 1 to 3 (CIN1, CIN2, and CIN3) according to the Bethesda classification (51). Participants who had received treatment for vaginal or urinary infections, who were currently pregnant or up to 2 months postpartum, who had a history of hysterectomy, or who had a severe chronic disease were excluded from the study.

Besides SIL and HPV status, the following study variables were included in the analysis: age, HPV subtype, smoking (daily number of cigarettes consumed at time of study), regularity of menstrual period, intermenstrual bleeding, current sexual activity status, use and type of contraceptives (IUD [all copper], tubal ligation, hormonal contraceptives, or condoms), vaginal douching, age of sexual debut, number of sexual partners, number of pregnancies, and number of births and miscarriages.

### Samples of vaginal exudate.

Samples of vaginal exudate were taken by swabbing the mucosa using sterile Teflon swabs that were placed in a sterile 15-ml conical plastic tube with sterile 0.9% sodium chloride (Baxter physiological saline solution). The sample was kept at −20°C until its use for microbiome sequencing analysis.

### Cervical DNA extraction and HPV detection and typing.

Cervical DNA was extracted directly from the brushing done by the gynecologist before the application of acetic acid for the colposcopy study. The brush was stored in 1 ml of saline solution at 4°C for transport. DNA was extracted immediately from a first centrifugation at 8,000 rpm for 5 min, and the pellet was resuspended in 200 μl of extraction buffer (SDS 1%) and proteinase K (200 μg/ml), making a modification in the digestion of the sample (53°C for 5 h) and inactivating the reaction by boiling for 5 min (52). Once the sample was digested and inactivated, it was centrifuged at 12,000 rpm for 5 min, and the supernatant was removed and frozen at −20°C until use. HPV was detected via PCR using two sets of oligonucleotides, MY09/MY11 (53) and GP5/GP6 (54). Cycling conditions were used as previously described for the detection of HPV DNA in cervical cells (53–55). HPV DNA obtained from HeLa cell cultures containing 10 to 50 copies of the HPV-18 open reading frame (ORF) L1 was used as a positive control (56). All positive samples for HPV were subsequently typed with the HPVFast 2.0 kit (Pharma Gen SA, Madrid, Spain) according to the manufacturer’s instructions.

### 16S rRNA gene sequencing.

From vaginal DNA samples, the 16S rRNA gene was amplified by PCR in triplicate using bar-coded primer pairs flanking the V3 region as previously described (57). Each 50-μl sample of PCR mixture contained 22 μl of water, 25 μl of TopTaq master mix (Qiagen), 0.5 μl of each forward and reverse bar-coded primer, and 2 μl of template DNA. The PCR program consisted of an initial DNA denaturation step at 95°C (5 min), 25 cycles of DNA denaturation at 95°C (1 min), an annealing step at 50°C (1 min), an elongation step at 72°C (1 min), and a final elongation step at 72°C (7 min). Controls without template DNA were included to ensure that no contamination occurred. Amplicons were run on a 2% agarose gel to ensure adequate amplification. Amplicons displaying bands at ∼160 base pairs (bp) were purified using an Illustra GFX PCR DNA purification kit. Purified samples were diluted 1:50 and quantified using PicoGreen (Invitrogen) in a Tecan M200 plate reader (excitation at 480 nm and emission at 520 nm).

For 16S rRNA gene sequencing, each PCR pool was analyzed on an Agilent Bioanalyzer using a high-sensitivity double-stranded DNA (dsDNA) assay to determine approximate library fragment size and verify library integrity. Pooled library concentrations were determined using the TruSeq DNA sample preparation kit, version 2 (Illumina). Library pools were diluted to 4 nM and denatured into single strands using fresh 0.2 N NaOH. The final library loading concentration was 8 pM, with an additional PhiX spike-in of 20%. Sequencing was performed using a Hi-Seq 2000 bidirectional Illumina sequencing and cluster kit, version 4 (Macrogen, Inc.). PCR products were visualized on E-gels, quantified using an Invitrogen Qubit with PicoGreen, and pooled at equal concentrations, according to a previous report (58).

### Bioinformatic analysis of 16S rRNA gene sequences.

All sequences were processed using mothur v.1.43.0 according to the standard operating procedure as previously described (59). Quality sequences were obtained by removing sequences with ambiguous bases, with a low-quality read length, and/or chimeras identified using chimera uchime. Quality sequences were aligned and compared to the SILVA bacterial reference version 132, and OTUs were generated using a dissimilarity cutoff of 0.03. The sequences were classified using the classify seqs command. There was a total of 1,099,273 reads in this sequencing run, which decreased to 1,072,800 after removing singletons and those samples with less than 1,000 reads with a median of 4,720 reads per sample (IQR of 1,175). A cutoff of 1,000 reads per sample was applied, and 63 were excluded from the analysis, yielding a final *N* of 228. Further filtering was applied to remove mitochondrion and chloroplast sequences and OTUs present in fewer than three samples. The OTU table was included as a supplemental data set in the supplemental material.

### Statistical analysis.

Differences in frequencies for categorical variables between cases and controls were evaluated using the chi-square statistic with Yates correction. Risk was estimated and expressed as an odds ratio (OR) and a 95% confidence interval (CI). For numerical variables, Mann-Whitney (nonparametric) or Student *t* tests (parametric) were used based on the D´Agostino-Pearson normality test. We assessed vaginal microbial diversity and the relative abundance of bacterial taxa using Phyloseq (60) along with additional R-based computational tools in R Studio (R Studio, Boston, MA). Principal-coordinate analysis (PCoA) was conducted using Phyloseq and was statistically confirmed by permutational multivariate analysis of variance (PERMANOVA; Adonis test). The Chao1 and Shannon diversity indices were calculated using Phyloseq and statistically confirmed by Mann-Whitney or Kruskal-Wallis and Dunn tests if more than two groups were compared (GraphPad Prism software, version 5c, San Diego, CA). Multivariate association with linear models (MaAsLin; see reference 61) were used to calculate differentially abundant OTUs between the cases and controls, including several other study variables available from the metadata. The following covariates were fitted into the MaAsLin model based on previously reported associations with HPV infection or with microbiome shifts: SIL grade, HPV infection, HPV type, current smoking status, intermenstrual bleeding, sexual activity status, use of contraceptives, type of contraceptive, genital hygiene, age, age of sexual debut, number of sexual partners, number of sexual partners by age, number of pregnancies, number of births, and number of miscarriages.

### Data availability.

Sequences have been deposited to BioProject (accession number PRJNA766648).

## References

[1] Berti FCB, Salviano-Silva A, Beckert HC, de Oliveira KB, Cipolla GA, Malheiros D. 2019. From squamous intraepithelial lesions to cervical cancer: circulating microRNAs as potential biomarkers in cervical carcinogenesis. Biochim Biophys Acta Rev Cancer 1872:188306. doi:10.1016/j.bbcan.2019.08.001. 31398380

[2] Borgogna JC, Shardell MD, Santori EK, Nelson TM, Rath JM, Glover ED, Ravel J, Gravitt PE, Yeoman CJ, Brotman RM. 2020. The vaginal metabolome and microbiota of cervical HPV-positive and HPV-negative women: a cross-sectional analysis. BJOG 127:182–192. doi:10.1111/1471-0528.15981. 31749298 PMC6982399

[3] Curty G, Costa RL, Siqueira JD, Meyrelles AI, Machado ES, Soares EA, Soares MA. 2017. Analysis of the cervical microbiome and potential biomarkers from postpartum HIV-positive women displaying cervical intraepithelial lesions. Sci Rep 7:17364. doi:10.1038/s41598-017-17351-9. 29234019 PMC5727204

[4] Wilkinson EJ, Cox JT, Selim MA, O’Connor DM. 2015. Evolution of terminology for human-papillomavirus-infection-related vulvar squamous intraepithelial lesions. J Low Genit Tract Dis 19:81–87. doi:10.1097/LGT.0000000000000049. 24832173

[5] Forman D, de Martel C, Lacey CJ, Soerjomataram I, Lortet-Tieulent J, Bruni L, Vignat J, Ferlay J, Bray F, Plummer M, Franceschi S. 2012. Global burden of human papillomavirus and related diseases. Vaccine 30 Suppl 5:F12–F23. doi:10.1016/j.vaccine.2012.07.055. 23199955

[6] Parkin DM, Almonte M, Bruni L, Clifford G, Curado MP, Piñeros M. 2008. Burden and trends of type-specific human papillomavirus infections and related diseases in the Latin America and Caribbean region. Vaccine 26 Suppl 11:L1–L15. doi:10.1016/j.vaccine.2008.05.043. 18945399

[7] Pérez-Quintanilla M, Méndez-Martínez R, Vázquez-Vega S, Espinosa-Romero R, Sotelo-Regil R, Pérez-Montiel MD, Ramos-Alamillo U, De Jesús Cabrera-López T, Barquet-Muñoz SA, Pérez-Plascencia C, García-Carrancá A, De León DC. 2020. High prevalence of human papillomavirus and European variants of HPV 16 infecting concomitantly to cervix and oral cavity in HIV positive women. PLoS One 15:e0227900. doi:10.1371/journal.pone.0227900. 32320400 PMC7176371

[8] Feng RM, Hu SY, Zhao FH, Zhang R, Zhang X, Wallach AI, Qiao YL. 2017. Role of active and passive smoking in high-risk human papillomavirus infection and cervical intraepithelial neoplasia grade 2 or worse. J Gynecol Oncol 28:e47. doi:10.3802/jgo.2017.28.e47. 28657217 PMC5540715

[9] Audirac-Chalifour A, Torres-Poveda K, Bahena-Román M, Téllez-Sosa J, Martínez-Barnetche J, Cortina-Ceballos B, López-Estrada G, Delgado-Romero K, Burguete-García AI, Cantú D, García-Carrancá A, Madrid-Marina V. 2016. Cervical microbiome and cytokine profile at various stages of cervical cancer: a pilot study. PLoS One 11:e0153274. doi:10.1371/journal.pone.0153274. 27115350 PMC4846060

[10] Vonaesch P, Anderson M, Sansonetti PJ. 2018. Pathogens, microbiome and the host: emergence of the ecological Koch’s postulates. FEMS Microbiol Rev 42:273–292. doi:10.1093/femsre/fuy003. 29325027

[11] Moosa Y, Kwon D, de Oliveira T, Wong EB. 2020. Determinants of vaginal microbiota composition. Front Cell Infect Microbiol 10:467. doi:10.3389/fcimb.2020.00467. 32984081 PMC7492712

[12] Łaniewski P, Cui H, Roe DJ, Barnes D, Goulder A, Monk BJ, Greenspan DL, Chase DM, Herbst-Kralovetz MM. 2019. Features of the cervicovaginal microenvironment drive cancer biomarker signatures in patients across cervical carcinogenesis. Sci Rep 9:7333. doi:10.1038/s41598-019-43849-5. 31089160 PMC6517407

[13] Ilhan ZE, Łaniewski P, Thomas N, Roe DJ, Chase DM, Herbst-Kralovetz MM. 2019. Deciphering the complex interplay between microbiota, HPV, inflammation and cancer through cervicovaginal metabolic profiling. EBioMedicine 44:675–690. doi:10.1016/j.ebiom.2019.04.028. 31027917 PMC6604110

[14] Kim TK, Thomas SM, Ho M, Sharma S, Reich CI, Frank JA, Yeater KM, Biggs DR, Nakamura N, Stumpf R, Leigh SR, Tapping RI, Blanke SR, Slauch JM, Gaskins HR, Weisbaum JS, Olsen GJ, Hoyer LL, Wilson BA. 2009. Heterogeneity of vaginal microbial communities within individuals. J Clin Microbiol 47:1181–1189. doi:10.1128/JCM.00854-08. 19158255 PMC2668325

[15] Piyathilake CJ, Ollberding NJ, Kumar R, Macaluso M, Alvarez RD, Morrow CD. 2016. Cervical microbiota associated with higher grade cervical intraepithelial neoplasia in women infected with high-risk human papillomaviruses. Cancer Prev Res (Phila) 9:357–366. doi:10.1158/1940-6207.CAPR-15-0350. 26935422 PMC4869983

[16] Gao W, Weng J, Gao Y, Chen X. 2013. Comparison of the vaginal microbiota diversity of women with and without human papillomavirus infection: a cross-sectional study. BMC Infect Dis 13:271. doi:10.1186/1471-2334-13-271. 23758857 PMC3684509

[17] Kovachev SM. 2020. Cervical cancer and vaginal microbiota changes. Arch Microbiol 202:323–327. doi:10.1007/s00203-019-01747-4. 31659380

[18] Shigehara K, Kawaguchi S, Sasagawa T, Furubayashi K, Shimamura M, Maeda Y, Konaka H, Mizokami A, Koh E, Namiki M. 2011. Prevalence of genital *Mycoplasma*, *Ureaplasma*, *Gardnerella*, and human papillomavirus in Japanese men with urethritis, and risk factors for detection of urethral human papillomavirus infection. J Infect Chemother 17:487–492. doi:10.1007/s10156-010-0203-0. 21213011

[19] Ritu W, Enqi W, Zheng S, Wang J, Ling Y, Wang Y. 2019. Evaluation of the associations between cervical microbiota and HPV infection, clearance, and persistence in cytologically normal women. Cancer Prev Res (Phila) 12:43–56. doi:10.1158/1940-6207.CAPR-18-0233. 30463989

[20] Anahtar MN, Byrne EH, Doherty KE, Bowman BA, Yamamoto HS, Soumillon M, Padavattan N, Ismail N, Moodley A, Sabatini ME, Ghebremichael MS, Nusbaum C, Huttenhower C, Virgin HW, Ndung'u T, Dong KL, Walker BD, Fichorova RN, Kwon DS. 2015. Cervicovaginal bacteria are a major modulator of host inflammatory responses in the female genital tract. Immunity 42:965–976. doi:10.1016/j.immuni.2015.04.019. 25992865 PMC4461369

[21] Lamont RF, Sobel JD, Akins RA, Hassan SS, Chaiworapongsa T, Kusanovic JP, Romero R. 2011. The vaginal microbiome: new information about genital tract flora using molecular based techniques. BJOG 118:533–549. doi:10.1111/j.1471-0528.2010.02840.x. 21251190 PMC3055920

[22] Mortaki D, Gkegkes ID, Psomiadou V, Blontzos N, Prodromidou A, Lefkopoulos F, Nicolaidou E. 2020. Vaginal microbiota and human papillomavirus: a systematic review. J Turk Ger Gynecol Assoc 21:193–200. doi:10.4274/jtgga.galenos.2019.2019.0051. 31564082 PMC7495129

[23] Bychkovsky BL, Ferreyra ME, Strasser-Weippl K, Herold CI, De Lima Lopes G, Dizon DS, Schmeler KM, Del Carmen M, Randall TC, Nogueira-Rodrigues A, De Carvalho Calabrich AF, St Louis J, Vail CM, Goss PE. 2016. Cervical cancer control in Latin America: a call to action. Cancer 122:502–514. doi:10.1002/cncr.29813. 26670695

[24] Fosch SE, Ficoseco CA, Marchesi A, Cocucci S, Nader-Macias MEF, Perazzi BE. 2018. Contraception: influence on vaginal microbiota and identification of vaginal lactobacilli using MALDI-TOF MS and 16S rDNA sequencing. Open Microbiol J 12:218–229. doi:10.2174/1874285801812010218. 30069261 PMC6047200

[25] Romero-Morelos P, Bandala C, Jiménez-Tenorio J, Valdespino-Zavala M, Rodríguez-Esquivel M, Gama-Ríos RA, Bandera A, Mendoza-Rodríguez M, Taniguchi K, Marrero-Rodríguez D, López-Romero R, Ramón-Gallegos E, Salcedo M. 2019. Vaginosis-associated bacteria and its association with HPV infection. Med Clin (Barc) 152:1–5. doi:10.1016/j.medcli.2018.01.027. 29544661

[26] Musa J, Achenbach CJ, O’Dwyer LC, Evans CT, McHugh M, Hou L, Simon MA, Murphy RL, Jordan N. 2017. Effect of cervical cancer education and provider recommendation for screening on screening rates: a systematic review and meta-analysis. PLoS One 12:e0183924. doi:10.1371/journal.pone.0183924. 28873092 PMC5584806

[27] Ferlay J, Colombet M, Soerjomataram I, Mathers C, Parkin DM, Piñeros M, Znaor A, Bray F. 2019. Estimating the global cancer incidence and mortality in 2018: GLOBOCAN sources and methods. Int J Cancer 144:1941–1953. doi:10.1002/ijc.31937. 30350310

[28] Wang R, Pan W, Jin L, Huang W, Li Y, Wu D, Gao C, Ma D, Liao S. 2020. Human papillomavirus vaccine against cervical cancer: opportunity and challenge. Cancer Lett 471:88–102. doi:10.1016/j.canlet.2019.11.039. 31812696

[29] Chan CK, Aimagambetova G, Ukybassova T, Kongrtay K, Azizan A. 2019. Human papillomavirus infection and cervical cancer: epidemiology, screening, and vaccination—review of current perspectives. J Oncol 2019:3257939. doi:10.1155/2019/3257939. 31687023 PMC6811952

[30] Illades-Aguiar B, del Alarcón-Romero LC, Antonio-Véjar V, Zamudio-López N, Sales-Linares N, Flores-Alfaro E, Fernández-Tilapa G, Vences-Velázquez A, Muñoz-Valle JF, Leyva-Vázquez MA. 2010. Prevalence and distribution of human papillomavirus types in cervical cancer, squamous intraepithelial lesions, and with no intraepithelial lesions in women from Southern Mexico. Gynecol Oncol 117:291–296. doi:10.1016/j.ygyno.2010.01.036. 20199804

[31] Zhang J, Cheng K, Wang Z. 2020. Prevalence and distribution of human papillomavirus genotypes in cervical intraepithelial neoplasia in China: a meta-analysis. Arch Gynecol Obstet 302:1329–1337. doi:10.1007/s00404-020-05787-w. 32914222 PMC7584548

[32] Castellsagué X. 2008. Natural history and epidemiology of HPV infection and cervical cancer. Gynecol Oncol 110:S4–S7. doi:10.1016/j.ygyno.2008.07.045. 18760711

[33] Cortessis VK, Barrett M, BrownWade N, Enebish T, Perrigo JL, Tobin J, Zhong C, Zink J, Isiaka V, Muderspach LI, Natavio M, McKean-Cowdin R. 2017. Intrauterine device use and cervical cancer risk: a systematic review and meta-analysis. Obstet Gynecol 130:1226–1236. doi:10.1097/AOG.0000000000002307. 29112647

[34] Agenjo González M, Lampaya Nasarre B, Salazar F, Varillas D, Cristobal I. 2019. Influence of intrauterine dispositive in human papillomavirus clearance. Eur J Obstet Gynecol Reprod Biol 232:65–69. doi:10.1016/j.ejogrb.2018.11.016. 30472624

[35] Chao XP, Sun TT, Wang S, Fan QB, Shi HH, Zhu L, Lang JH. 2019. Correlation between the diversity of vaginal microbiota and the risk of high-risk human papillomavirus infection. Int J Gynecol Cancer 29:28–34. doi:10.1136/ijgc-2018-000032. 30640680

[36] Huang SI, Chen J, Lian LY, Cai HH, Zeng HS, Zeng M, Liu MB. 2020. Intratumoral levels and prognostic significance of *Fusobacterium nucleatum* in cervical carcinoma. Aging (Albany NY) 12:23337–23350. doi:10.18632/aging.104188. 33197886 PMC7746363

[37] Hu J, Gao Y, Zheng Y, Shang X. 2018. KF-finder: identification of key factors from host-microbial networks in cervical cancer. BMC Syst Biol 12:54. doi:10.1186/s12918-018-0566-x. 29745858 PMC5998879

[38] Wudtisan J, Tantipalakorn C, Charoenkwan K, Sreshthaputra RA, Srisomboon J. 2019. Factors associated with development of high-grade squamous intraepithelial lesions of the uterine cervix in women younger than 30 years. Asian Pac J Cancer Prev 20:1031–1036. doi:10.31557/APJCP.2019.20.4.1031. 31030470 PMC6948903

[39] Pelkofski E, Stine J, Wages NA, Gehrig PA, Kim KH, Cantrell LA. 2016. Cervical cancer in women aged 35 years and younger. Clin Ther 38:459–466. doi:10.1016/j.clinthera.2016.01.024. 26899314

[40] Jespers V, Hardy L, Buyze J, Loos J, Buvé A, Crucitti T. 2016. Association of sexual debut in adolescents with microbiota and inflammatory markers. Obstet Gynecol 128:22–31. doi:10.1097/AOG.0000000000001468. 27275789

[41] Martino JL, Vermund SH. 2002. Vaginal douching: evidence for risks or benefits to women’s health. Epidemiol Rev 24:109–124. doi:10.1093/epirev/mxf004. 12762087 PMC2567125

[42] Łaniewski P, Barnes D, Goulder A, Cui H, Roe DJ, Chase DM, Herbst-Kralovetz MM. 2018. Linking cervicovaginal immune signatures, HPV and microbiota composition in cervical carcinogenesis in non-Hispanic and Hispanic women. Sci Rep 8:7593. doi:10.1038/s41598-018-25879-7. 29765068 PMC5954126

[43] Devillard E, Burton JP, Reid G. 2005. Complexity of vaginal microflora as analyzed by PCR denaturing gradient gel electrophoresis in a patient with recurrent bacterial vaginosis. Infect Dis Obstet Gynecol 13:25–30. doi:10.1155/2005/607474. 16040324 PMC1784553

[44] Ling Z, Liu X, Chen X, Zhu H, Nelson KE, Xia Y, Li L, Xiang C. 2011. Diversity of cervicovaginal microbiota associated with female lower genital tract infections. Microb Ecol 61:704–714. doi:10.1007/s00248-011-9813-z. 21287345

[45] Antonio MAD, Meyn LA, Murray PJ, Busse B, Hillier SL. 2009. Vaginal colonization by probiotic *Lactobacillus crispatus* CTV-05 is decreased by sexual activity and endogenous lactobacilli. J Infect Dis 199:1506–1513. doi:10.1086/598686. 19331578

[46] Borgdorff H, Armstrong SD, Tytgat HLP, Xia D, Ndayisaba GF, Wastling JM, van de Wijgert JHHM. 2016. Unique insights in the cervicovaginal *Lactobacillus iners* and *L. crispatus* proteomes and their associations with microbiota dysbiosis. PLoS One 11:e0150767. doi:10.1371/journal.pone.0150767. 26963809 PMC4786256

[47] Borgdorff H, Gautam R, Armstrong SD, Xia D, Ndayisaba GF, van Teijlingen NH, Geijtenbeek TBH, Wastling JM, van De Wijgert JHHM. 2016. Cervicovaginal microbiome dysbiosis is associated with proteome changes related to alterations of the cervicovaginal mucosal barrier. Mucosal Immunol 9:621–633. doi:10.1038/mi.2015.86. 26349657

[48] Ma B, Forney LJ, Ravel J. 2012. Vaginal microbiome: rethinking health and disease. Annu Rev Microbiol 66:371–389. doi:10.1146/annurev-micro-092611-150157. 22746335 PMC3780402

[49] Ravel J, Gajer P, Abdo Z, Schneider GM, Koenig SSK, McCulle SL, Karlebach S, Gorle R, Russell J, Tacket CO, Brotman RM, Davis CC, Ault K, Peralta L, Forney LJ. 2011. Vaginal microbiome of reproductive-age women. Proc Natl Acad Sci USA 108 (Suppl 1):4680–4687. doi:10.1073/pnas.1002611107. 20534435 PMC3063603

[50] White BA, Creedon DJ, Nelson KE, Wilson BA. 2011. The vaginal microbiome in health and disease. Trends Endocrinol Metab 22:389–393. doi:10.1016/j.tem.2011.06.001. 21757370 PMC3183339

[51] Berek JS. 2003. Simplification of the new Bethesda 2001 classification system. Am J Obstet Gynecol 188:S2–S5. doi:10.1067/mob.2003.220. 12634623

[52] Sepp R, Szabo I, Uda H, Sakamoto H. 1994. Rapid techniques for DNA extraction from routinely processed archival tissue for use in PCR. J Clin Pathol 47:318–323. doi:10.1136/jcp.47.4.318. 8027368 PMC501934

[53] Manos MM, Ting YT, Wright DK, Lewis AL, Broker TR, Wolinsky SM, Manos M, Ting YC. 1989. The use of polymerase chain reaction amplification for the detection of genital human papillomaviruses. Cancer Cells 7:209–214. https://www.scienceopen.com/document?vid=9ec5d1ec-05b8-486f-aa20-7279b7add122.

[54] de Roda Husman AM, Walboomers JM, van den Brule AJ, Meijer CJ, Snijders PJ. 1995. The use of general primers GP5 and GP6 elongated at their 3′ ends with adjacent highly conserved sequences improves human papillomavirus detection by PCR. J Gen Virol 76 (Pt 4):1057–1062. doi:10.1099/0022-1317-76-4-1057. 9049358

[55] Liaw KL, Glass AG, Manos MM, Greer CE, Scott DR, Sherman M, Burk RD, Kurman RJ, Wacholder S, Rush BB, Cadell DM, Lawler P, Tabor D, Schiffman M. 1999. Detection of human papillomavirus DNA in cytologically normal women and subsequent cervical squamous intraepithelial lesions. J Natl Cancer Inst 91:954–960. doi:10.1093/jnci/91.11.954. 10359548

[56] Meissner JD. 1999. Nucleotide sequences and further characterization of human papillomavirus DNA present in the CaSki, SiHa and HeLa cervical carcinoma cell lines. J Gen Virol 80 (Pt 7):1725–1733. doi:10.1099/0022-1317-80-7-1725. 10423141

[57] Bartram AK, Lynch MDJ, Stearns JC, Moreno-Hagelsieb G, Neufeld JD. 2011. Generation of multimillion-sequence 16S rRNA gene libraries from complex microbial communities by assembling paired-end Illumina reads. Appl Environ Microbiol 77:3846–3852. doi:10.1128/AEM.02772-10. 21460107 PMC3127616

[58] Comeau AM, Douglas GM, Langille MGI. 2017. Microbiome helper: a custom and streamlined workflow for microbiome research. mSystems 2:e00127-16. doi:10.1128/mSystems.00127-16. PMC5209531 28066818

[59] Schloss PD, Gevers D, Westcott SL. 2011. Reducing the effects of PCR amplification and sequencing artifacts on 16s rRNA-based studies. PLoS One 6:e27310. doi:10.1371/journal.pone.0027310. 22194782 PMC3237409

[60] McMurdie PJ, Holmes S. 2015. Shiny-phyloseq: web application for interactive microbiome analysis with provenance tracking. Bioinformatics 31:282–283. doi:10.1093/bioinformatics/btu616. 25262154 PMC4287943

[61] Morgan XC, Tickle TL, Sokol H, Gevers D, Devaney KL, Ward DV, Reyes JA, Shah SA, LeLeiko N, Snapper SB, Bousvaros A, Korzenik J, Sands BE, Xavier RJ, Huttenhower C. 2012. Dysfunction of the intestinal microbiome in inflammatory bowel disease and treatment. Genome Biol 13:R79. doi:10.1186/gb-2012-13-9-r79. 23013615 PMC3506950

